# Variant evolution graph: Can we infer how SARS-CoV-2 variants are evolving?

**DOI:** 10.1371/journal.pone.0323970

**Published:** 2025-06-09

**Authors:** Badhan Das, Lenwood S. Heath

**Affiliations:** Department of Computer Science, Virginia Polytechnic Institute and State University, Blacksburg, Virginia, United States of America; Politecnico di Milano, ITALY

## Abstract

The SARS-CoV-2 virus has undergone extensive mutations over time, resulting in considerable genetic diversity among circulating strains. This diversity directly affects important viral characteristics, such as transmissibility and disease severity. During a viral outbreak, the rapid mutation rate produces a large cloud of variants, referred to as a viral quasispecies. However, many variants are lost due to the bottleneck of transmission and survival. Advances in next-generation sequencing have enabled continuous and cost-effective monitoring of viral genomes, but constructing reliable phylogenetic trees from the vast collection of sequences in GISAID (the Global Initiative on Sharing All Influenza Data) presents significant challenges.

We introduce a novel graph-based framework inspired by quasispecies theory, the Variant Evolution Graph (VEG), to model viral evolution. Unlike traditional phylogenetic trees, VEG accommodates multiple ancestors for each variant and maps all possible evolutionary pathways. The strongly connected subgraphs in the VEG reveal critical evolutionary patterns, including recombination events, mutation hotspots, and intra-host viral evolution, providing deeper insights into viral adaptation and spread. We also derive the Disease Transmission Network (DTN) from the VEG, which supports the inference of transmission pathways and super-spreaders among hosts.

We have applied our method to genomic data sets from five arbitrarily selected countries — Somalia, Bhutan, Hungary, Iran, and Nepal. Our study compares three methods for computing mutational distances to build the VEG, sourmash, pyani, and edit distance, with the phylogenetic approach using Maximum Likelihood (ML). Among these, ML is the most computationally intensive, requiring multiple sequence alignment and probabilistic inference, making it the slowest. In contrast, sourmash is the fastest, followed by the edit distance approach, while pyani takes more time due to its BLAST-based computations. This comparison highlights the computational efficiency of VEG, making it a scalable alternative for analyzing large viral data sets.

## Introduction

The COVID-19 pandemic was caused by severe acute respiratory syndrome coronavirus 2 (SARS-CoV-2), a beta-coronavirus closely related to the human SARS-CoV virus responsible for the SARS outbreak of 2002-2004 [1]. SARS-CoV-2 has caused millions of deaths and significant global health impacts. The pandemic has produced an unprecedented volume of genetic data for a single pathogen [2], significantly contributing to efforts in understanding the biology of the virus and combating its spread. It has also allowed direct observation of evolutionary processes that were previously inferred indirectly, such as the diversification of SARS-CoV-2 into distinct variants with varying phenotypic traits, including differences in transmissibility, disease severity, and immune evasion [1].

RNA-dependent RNA polymerases (RdRps) are crucial enzymes for the replication of RNA viruses, characterized by their lack of proofreading capabilities, which leads to high mutation rates during viral replication. While some RNA viruses, including coronaviruses, possess a weak proofreading exonuclease (ExoN) protein, the overall absence of efficient error correction significantly influences their evolutionary dynamics. This absence of proofreading enables RNA viruses to adapt to environmental pressures rapidly, evade host immune responses, and develop resistance to antiviral therapies. The high mutation rates associated with RdRps are primarily due to their inherent biochemical properties. Unlike DNA polymerases, which possess proofreading mechanisms that enhance replication fidelity, RdRps typically introduce errors at a rate of approximately 1 in 10,000 nucleotides copied, resulting in a mutation rate of about 10^−4^ mutations per nucleotide [3,4]. This high error frequency is further exacerbated by the lack of $3^{\prime }$ to $5^{\prime }$ exonuclease activity, a feature present in many DNA polymerases that corrects misincorporated nucleotides [5,6]. Consequently, a diverse population of closely related viral genomes emerges, collectively known as a quasispecies. This genetic diversity enables rapid adaptation to environmental pressures, such as host immune responses or antiviral treatments [7,8]. The implications of this high mutation rate are profound. For instance, the ability of RNA viruses to rapidly mutate enables them to escape immune detection and adapt to new hosts or therapeutic interventions [9]. This adaptability poses significant challenges for vaccine development and antiviral strategies, as the viral population can quickly shift in response to selective pressures [10]. Additionally, the formation of quasispecies can lead to increased virulence and pathogenicity, as certain variants may possess enhanced fitness traits that allow them to dominate the viral population [4,8]. Furthermore, while some RNA viruses, such as coronaviruses, have evolved additional mechanisms to mitigate the effects of high mutation rates, such as employing an exonuclease for proofreading, most RNA viruses rely solely on their RdRps, which lack such capabilities [11,12]. This evolutionary trade-off highlights the balance between rapid replication and fidelity, a critical aspect of RNA virus biology that influences their epidemiology and the development of effective treatments [5,6].

Coronaviruses, like other RNA viruses, develop rapidly, usually over months or years, with evident and quantitative outcomes. Evolution happens in periods that correspond to viral transmission events and ecological processes. As a result, evolutionary, ecological, and epidemiological processes impact each other in studying RNA viruses [13]. Viral evolution is influenced by how mutations are generated and transmitted within populations [14]. Natural selection will fix favorable mutations, such as the D614G mutation, which increases transmissibility [15]. Viral evolution adds another layer of complexity, since viruses multiply and evolve within individuals while effectively transmitting from person to person, resulting in evolution on a different scale. Most variation is lost during the tight bottlenecks imposed at transmission, but other changes are often passed on by chance without selective benefit [16]. In addition to population-level dynamics, when viral lineages evolve, possibly resulting in antigenically different strains, higher-level processes such as lineage competition and extinction arise [1].

A phylogenetic tree or network is commonly used to illustrate the evolutionary history of a group of species, and this model has immensely aided in hypothesis development and testing. The SARS-CoV-2 genomic data set constantly grows as additional genomes are sequenced [17]. This expansion of the data set means that the phylogeny of SARS-CoV-2 must regularly be updated [18], and the size of these data poses significant computational challenges for complete phylogenetic analysis [17]. According to Morel *et al*. [19], it is difficult to infer a reliable phylogeny on the GISAID data due to the enormous number of sequences and the small number of mutations. Furthermore, rooting the predicted phylogeny with some confidence, either through the bat and pangolin out-groups or by applying fresh computational methods to the in-group phylogeny, does not appear to be feasible [19]. Additionally, employing different multiple sequence alignment (MSA) strategies impacts the result of downstream phylogenetic analyses [20–22].

Viral quasispecies, also known as mutant spectra, swarms, or clouds, occur during the reproduction of RNA viruses in infected hosts [23,24]. The concept of quasispecies stems from a speculative molecular evolution model that highlights the importance of error-prone, complex, and dynamic replication of primary RNA or RNA-like replicons in early life forms’ self-organization and flexibility [25,26]. Quasispecies theory outlines the development of an infinite population of asexual replicators with high mutation rates [27]. Due to that, the commonly utilized model for understanding viral evolution in a host is the theory of quasispecies [28,29].

The overall representation of viral evolution differs significantly from a traditional phylogenetic tree or network due to the nature of viral populations. Unlike phylogenetic models, where new variants emerge as distinct branches, viral evolution often involves the coexistence of ancestral and newly evolved variants within the same population, forming a dynamic cloud of genetic diversity. To better capture this complexity, we introduce a graph-based data structure inspired by the quasispecies theory, a well-established model for viral evolution [23,29–36]. Quasispecies theory models viral populations as a continuously evolving dynamic system tightly linked to population dynamics [37]. In this context, a viral genotype is defined as a nucleotide sequence, and all possible genotypes form an exponentially large genome sequence space. However, most of these theoretically possible genotypes do not exist due to natural bottlenecks, such as constraints on transmission and survival. Only genotypes successfully overcoming these bottlenecks are found in infected hosts, driving the observed diversity in viral populations.

This paper introduces the Variant Evolution Graph (VEG), a novel framework for modeling viral evolution. The foundation of this approach is rooted in quasispecies theory, setting it apart from existing studies and offering a new avenue for future research. In VEG, vertices represent the genotypes of viral variants along with their associated hosts, while edges capture the mutational distance and evolutionary direction. Genotype evolution is modeled as paths within this graph. During a pandemic, it is crucial to have a comprehensive understanding of the pathogen’s dynamics, and real-time tracking of its evolution can help uncover its mechanisms, predict future mutation patterns, and inform preventative and treatment strategies [1]. VEG provides an overall view of viral evolution, highlighting how mutations connect variants. Additionally, we propose a Disease Transmission Network (DTN) derived from VEG, which reveals transmission pathways among hosts in a specific location, offering insights into the local epidemiological landscape. VEG and DTN present a comprehensive model that captures both viral evolution and transmission dynamics. Following the isolation protocol, we created location-specific genome data sets by downloading sequences from GISAID, grouped by country. Five countries were selected arbitrarily for this study: Somalia (22 sequences), Bhutan (102 sequences), Hungary (581 sequences), Iran (1,334 sequences), and Nepal (1,719 sequences). These location-based data sets were then used to run our proposed algorithm, generating both the Variant Evolution Graph (VEG) and the Disease Transmission Network (DTN).

## Preliminaries

### SARS-CoV-2 viral genome

The emergence of SARS-CoV-2 led to a global pandemic [38]. Coronaviruses are enveloped, single-stranded RNA viruses with genomes approximately 29,903 base pairs in length [39]. This RNA genome encodes multiple genes responsible for critical functions such as replication, transcription, and host infection. According to GISAID, the SARS-CoV-2 genome contains fifteen key genes listed in Table 1.

**Table 1:** Fifteen key genes of the SARS-CoV-2 genome and their locations in the reference genome of SARS-CoV-2 isolate Wuhan-Hu-1 [40].

Gene	Location
ORF1ab [40–42]	266..21555
ORF1a [40–42]	266..13468
S [41,42]	21563..25384
ORF3a [41,42]	25393..26220
ORF3b [41,42]	25765..26220
E	26245..26472
M [41,42]	26523..27191
ORF6 [41,42]	27202..27387
ORF7a [41,42]	27394..27759
ORF7b [41,42]	27756..27887
ORF8 [41,42]	27894..28259
N [41,42]	28274..29533
ORF9b [41,42]	28284..28577
ORF9c [41]	28734..28955
ORF10	29558..29674

### Average nucleotide identity

Average nucleotide identity (ANI) is a widely used measure that assesses nucleotide-level genomic similarity between the coding regions of two genomes [43]. Introduced by Goris *et al*. (2007) as a computational alternative to traditional DNA-DNA hybridization (DDH), ANI was designed to mimic the DDH experimental approach [44]. The resulting similarity score ranges from 0 to 1 and is typically reported as a percentage. An ANI value of 95% to 96% is generally considered equivalent to the historical 70% DDH threshold used to delineate species, making ANI a standard tool in microbial taxonomy [45].

#### Sourmash.

Sourmash is a software tool for comparing and analyzing extensive collections of genomes or other biological sequences, such as transcriptomes or metagenomes [46,47]. It compares sequences based on *k*-mer content, which are short, fixed-length substrings of sequences. MinHash is a technique for generating signatures and compact representations of *k*-mer contents in sourmash. These signatures are generated using hashing techniques and contain information about the presence and abundance of specific *k*-mer within the sequences. Fractional MinHash or FracMinHash is a refined technique used to create more compact and efficient MinHash sketches while maintaining the accuracy of similarity estimates. Sourmash (v4.4) can estimate ANI between two FracMinHash (scaled) sketches [48].

#### Pyani.

Pyani is a Python-based program and package designed to compute average nucleotide identity (ANI) and related measures for whole-genome comparisons, while also generating graphical and tabular summary outputs [49]. The tool offers four sub-commands for conducting ANI analyses: anim (ANIm, which utilizes the MUMmer software for sequence alignment), anib (ANIb, based on BLAST+), aniblastall (ANIb using the legacy BLAST tool), and tetra (which calculates genomic similarity using tetranucleotide frequency correlation coefficients). For our analysis, we used the anib sub-command.

### Levenshtein (edit) distance

Levenshtein distance, also referred to as edit distance, is a metric used to quantify the dissimilarity between two strings [50]. It is defined as the minimum number of edit operations required to transform one string into another. These operations include the substitution of one character for another, the deletion of a character, and the insertion of a character. Edit distance offers a quantitative approach for comparing and aligning sequences.

### Defining mutational distance

In this study, we employ two measures to represent mutational distance between genome sequences: edit distance and (1–*ANI*). Both are collectively referred to as mutational distance throughout the methodology for clarity and consistency. The edit distance directly quantifies the nucleotide-level changes required to transform one genome into another, thereby serving as a natural representation of genotypic divergence. In contrast, average nucleotide identity is inherently a similarity measure. To align it with the concept of dissimilarity, we compute (1–*ANI*), which quantifies the degree of difference between two genomes. Although (1–*ANI*) does not fulfill all formal mathematical distance metric properties, we adopt the term mutational distance for both measures to maintain consistency in our method descriptions.

### Introducing variant evolution graph

In a given location, consider a virus undergoing mutations and evolving. Within a specific period, *n* variants emerge, each associated with a collection (evolution) date. The Variant Evolution Graph, VEG=(V,E,w), is a directed acyclic graph where the vertex set *V* represents the viral strains. The edge set E={(u,v)∣v strain evolved from u} consists of directed, weighted edges that capture the mutational distances and evolutionary directions, reflecting parent-child relationships. The weight function w:E→ℝ assigns mutational distances to the edges, providing a quantitative representation of the evolutionary transitions within VEG.

### Problem statement

Let *L* be a genome space of the genotypes of *n* strains of that virus in a specific location associated with their collection dates, as follows:


$$L=\{g_{1},g_{2},...,g_{n}\}.$$

Here, *g*_*i*_ is the ${\text{i}}^{\text{th}}$ genome in set *L*, where the genomes are sorted in ascending order of their collection dates.

Let the set of collection dates of the genomes in *L* be $C=\{c_{1},c_{2},...,c_{n}\}$, where *c*_*i*_ is the collection date of *g*_*i*_. We define ϕ as the mapping from genomes in *L* to the set of collection dates, *C*,


ϕ:L→C

such that, $\phi (g_{i})=c_{i}$.

Let *D* be an n×n distance matrix, where *D*_*ij*_ is the mutational distance between the ${\text{i}}^{\text{th}}$ and ${\text{j}}^{\text{th}}$ genomes; 1≤i,j≤n, by some fixed measure of mutational distance. Here, the rows and columns of *D* are ordered in ascending order based on the collection dates of their corresponding variants.

Our first problem is as follows.

**Variant Evolution Graph**

**Instance:** A genome space *L* of size *n*, and an n×n distance matrix, *D*.

**Solution:** A directed acyclic graph, G=(V,E,w), based on minimum pair-wise mutational distances in *D*, where the weight of an edge (*u*,*v*) is $w(u,v)=D_{uv}$.

Our second problem:

**Disease Transmission Network**

**Instance:** A variant evolution graph, G=(V,E), set of collection dates, *C*, a mapping ϕ:V→C, and infection period of the virus, μ.

**Solution:** A directed acyclic graph, $T=(V,E^{\prime },w^{\prime })$, where $E^{\prime }=\{(u,v)|(u,v)\in E\text{, }|\phi (u)-\phi (v)|\leq \mu \}$ and the weight of an edge (*u*,*v*) is $w^{\prime }(u,v)=|\phi (u)-\phi (v)|$.

## Materials and methods

### Data set

For building the Variant Evolution Graph (VEG), we utilized the genotypes of SARS-CoV-2 variants. The complete reference genome of the SARS-CoV-2 isolate Wuhan-Hu-1 was downloaded from NCBI [40], and the genome sequences of the variants were obtained from GISAID, ensuring they were complete, high-coverage, and had complete collection dates. The genome space for constructing the graph was organized by location, either country-wise or state-wise, following the isolation protocol during the COVID-19 pandemic. Specifically, we worked with genomic data sets from Somalia, Bhutan, Hungary, Iran, and Nepal.

To manage genomes with ambiguous bases (Ns), we introduced a variable τ, representing the cumulative threshold for the percentage of Ns allowed in a data set. We set τ=0 for our experiments, meaning only genomes without Ns were included. This choice was made to ensure accurate mutational distance calculations and avoid the uncertainty introduced by ambiguous bases. However, this is not a strict requirement; users have the flexibility to set τ to any value (recommended to keep it below 1%) if they wish to include genomes with Ns based on their research needs. Table 2 shows the count of genomes before and after applying this filtering criterion.

**Table 2:** Table showing the counts of genomes before and after filtering based on τ=0 for Somalia, Bhutan, Hungary, Iran, and Nepal data sets.

Country	Count of genomes	
	Before filtering	After filtering
Somalia	22	10
Bhutan	102	65
Hungary	589	471
Iran	1334	821
Nepal	1719	616

### Edit distance calculation

**Fig 1:**
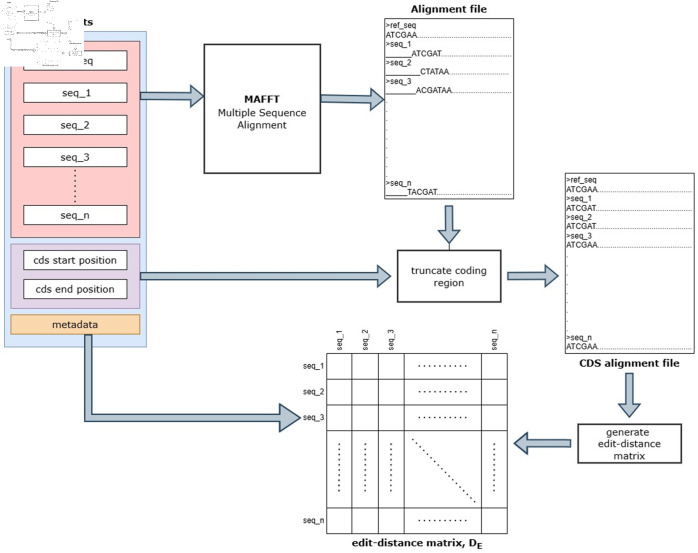
Pipeline for generating edit-distance matrix using pairwise edit distances between the variant genomes.

According to the positions of the genes, we considered positions 266 to 29674 as the coding region for the calculation of edit distance. The locations of these genes in the reference genome are given in Table 1. As shown in Fig 1, initially, we used MAFFT (Multiple Alignment using Fast Fourier Transform) v7.525 [51]. Following the alignment, the coding region in each sequence is extracted by truncating the sequences between the start and end positions. Focusing on the coding region ensures biological relevance, as it encompasses the functional regions of the genome where mutations are more likely to influence viral behavior. Additionally, this approach reduces noise by excluding non-coding regions, allowing for a more granular analysis of mutations with potential evolutionary and functional significance.

### Algorithms

#### Assumptions.

Our methods are based on the following assumptions:

1. The collection date of a given variant represents the time of its first appearance in the population.
2. Variants that share the same collection date are assumed not to have evolved from one another.

#### Computing distance matrix.

We applied sourmash and pyani to a set of genome sequences to generate two distance matrices: *D*_*S*_ and *D*_*P*_. Sourmash produces a distance matrix where each entry represents the dissimilarity between genome pairs, computed as 1–*ANI*. This matrix is denoted as *D*_*S*_. In contrast, pyani returns an ANI percentage identity matrix, *D*_*I*_, where each element represents the nucleotide-level similarity between genomes. Since we define mutational distance as the dissimilarity measure, 1–*ANI*, we convert the similarity matrix *D*_*I*_ into a distance matrix *D*_*P*_ using the relation $D_{P}=1-D_{I}$.

To compute the edit distance for the distance matrix, we focused on the coding region rather than the whole genome sequence. As shown in Fig 1, we used the truncated alignment file to compute pairwise edit distances. We calculated all the edit distances between pairwise genomes and generated the edit-distance matrix, *D*_*E*_.

#### Building the variant evolution graph.

To enforce temporal consistency, the genome set *L* is first sorted in ascending order based on collection dates, from the earliest to the most recent. The distance matrix *D* is then reordered accordingly to preserve alignment with this sorted genome list. Following the second assumption, we apply Algorithm 1 to refine *D*, ensuring that pairwise distances between genomes collected on the same date are excluded from further analysis.

**Algorithm 1. Organize-Distance-Matrix (*D*,*L*,*dates*).**



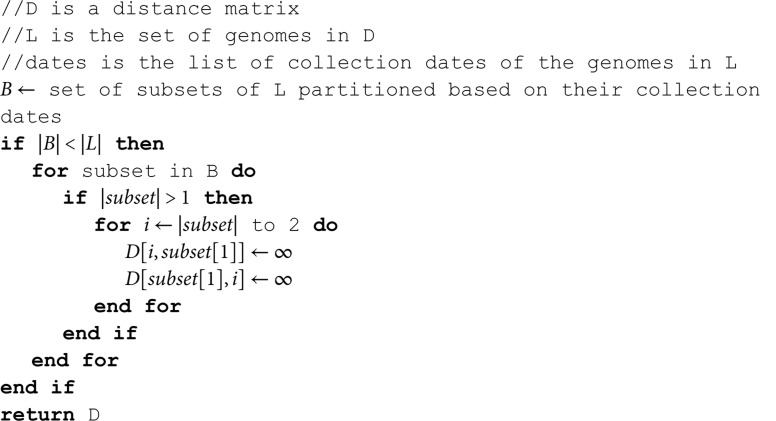



Given *n* variant genomes with *B* unique collection dates (B≤n), the genome set is partitioned into *B* groups, each corresponding to a distinct collection date. Since the genomes in *L* are sorted chronologically, the resulting groups are ordered from oldest to most recent. In this framework, variants in the ${\text{i}}^{\text{th}}$ group (1<i≤B) are allowed to evolve only from variants in earlier groups, reflecting the temporal constraint on evolutionary direction. The first group (i=1) represents the earliest collected variants and, therefore, contains no ancestral candidates. To enforce the second assumption—that variants with the same collection date cannot evolve from one another—Algorithm 1 sets all pairwise distances within each group to ∞, effectively excluding intra-group evolutionary relationships from consideration.

As detailed in Algorithm 2, the core objective of the method is to reconstruct evolutionary pathways by identifying, for each variant *v*, the closest ancestral variant(s) based on mutational distance. Specifically, for a given variant *v*, we define the set *P* to contain all variants with the minimum mutational distance to *v*. These variants in *P* are considered potential parents of *v*, and reciprocally, *v* is treated as a child of each variant in *P*.

The resulting parent-child relationships are used to construct a directed graph G=(V,E,w), where the vertex set *V* corresponds to the genome set *L*, and each edge (u,v)∈E represents a possible evolutionary link from variant *u* to *v*. The weight function *w* assigns each edge a value equal to the mutational distance $D_{uv}$, i.e., $w=\{D_{uv}|(u,v)\in E\}$.

**Fig 2:**
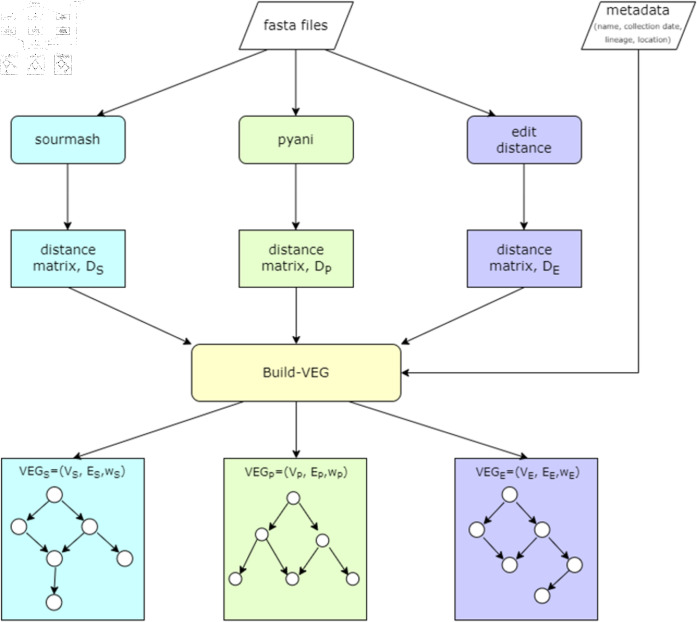
The whole pipeline of building VEG. The edit distance computation in this pipeline is separately shown in Fig 1.

This procedure produces a directed acyclic graph (DAG) rather than a strict tree, since a variant may have multiple equally distant ancestors. Evolutionary pathways are represented as directed paths within this graph. The whole pipeline for constructing the variant evolution graph (VEG) is illustrated in Fig 2. The Build-VEG method generates three such graphs: $G_{S}=(V_{S},E_{S},w_{S})$, $G_{P}=(V_{P},E_{P},w_{P})$, and $G_{E}=(V_{E},E_{E},w_{E})$, corresponding to distance matrices derived from sourmash, pyani, and edit distance, respectively.

**Algorithm 2. Build-VEG (*D*,*L*).**



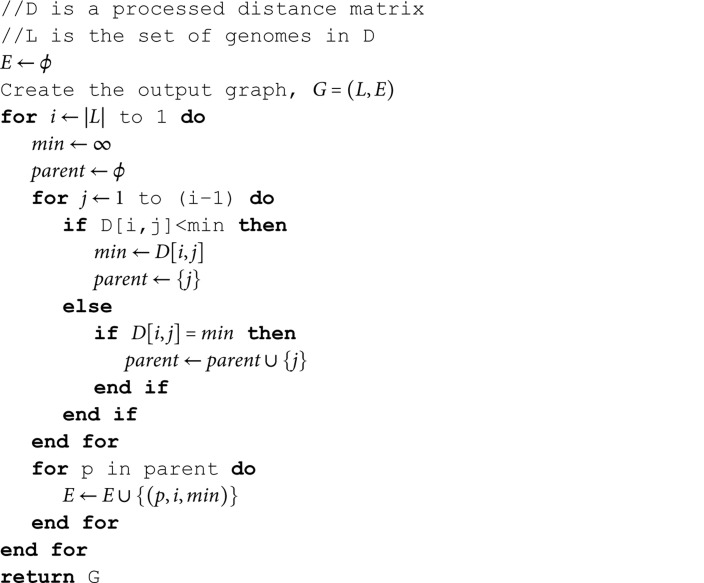



### An example

**Fig 3:**
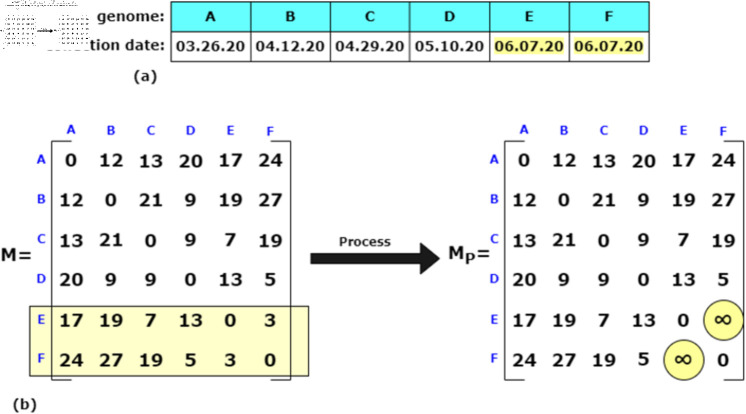
A sample set of six genomes. (a) The set of six variant genomes, *A*, *B*, *C*, *D*, *E*, and *F*, and their corresponding collection dates. (b) The workflow of Algorithm 1 on the distance matrix, *M*.

To illustrate the method, consider a simplified genome space consisting of six viral genomes: *A*, *B*, *C*, *D*, *E*, and *F*, as shown in Fig 3. Each genome is associated with a collection date, which, in accordance with Assumption 1, is treated as the date the variant first appeared in the population. The corresponding distance matrix, derived from sourmash, pyani, or edit distance, is sorted so that its rows and columns follow the ascending order of these collection dates.

In this example, genomes *E* and *F* share the same collection date (June 7, 2020). Based on Assumption 2, variants with identical collection dates cannot evolve from one another. Therefore, their mutual distances are set to infinity: $D_{EF}=\infty$ and $D_{FE}=\infty$, effectively excluding them from being considered in a direct evolutionary relationship. For this illustration, we use the edit distance matrix as the input.

**Fig 4:**
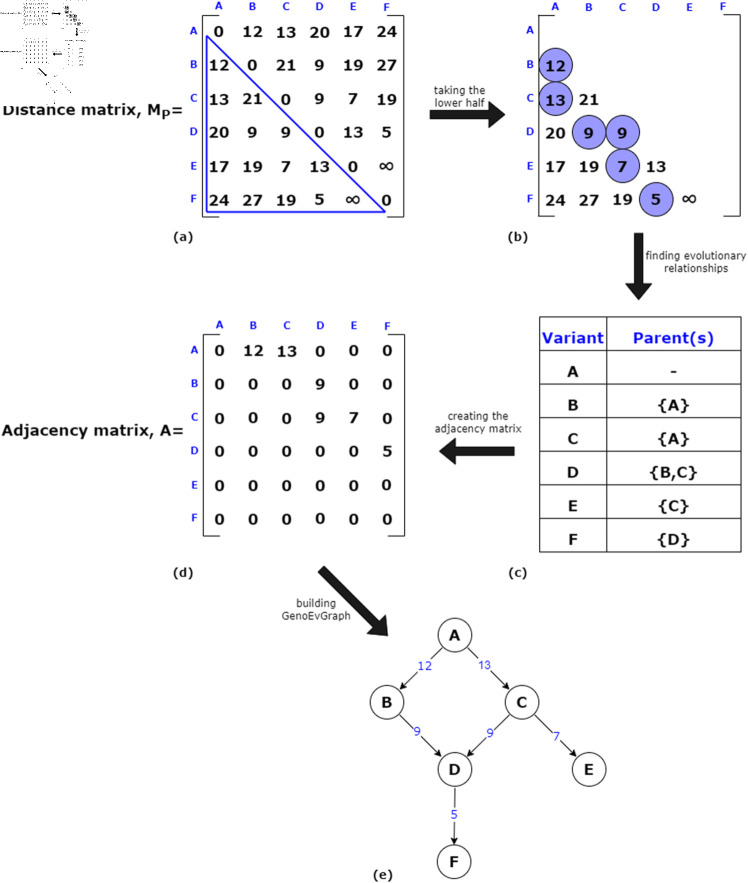
The workflow of Algorithm 2 with the example in Fig 3.

The sorted distance matrix is then provided as input to the Build-VEG algorithm, along with the genome set L={A,B,C,D,E,F}. For each genome v∈L, the algorithm identifies the minimal mutational distance(s) within the corresponding row of the distance matrix. These minimal distances, which indicate the closest potential parent(s) of a given variant, are visually highlighted in Fig 4 using blue circles.

In cases where multiple genomes share the same minimal distance to a given genome, multiple parent relationships are inferred. For example, genome *D* has a minimum distance value of 9 to both *B* and *C* ($D_{DB}=D_{DC}=9$), indicating that *D* has two inferred parents. After identifying the closest variants for each genome, we construct the corresponding parent sets and derive the evolutionary relationships accordingly.

Let $G_{E}=(V_{E},E_{E},w_{E})$ represent the resulting variant evolution graph (VEG) constructed using edit distance, where $V_{E}=L$. Since the procedure traces parent variants for each child genome, the relationships are reversed to form directed edges of the form (*u*,*v*), where *u* is a parent of *v*. The weight function *w*_*E*_ assigns each edge a value corresponding to the mutational distance, such that $w_{E}=\{D_{uv}|(u,v)\in E_{E}\}$.

### Deriving the distance transmission network

Since VEG is built using mutation data and the evolution timeline, it reveals the evolutionary relationships among strains within the genome space and helps infer the Disease Transmission Network (DTN) for a specific location, as each strain is linked to a patient or host. The edges of the VEG also capture the time differences between the collection dates of the strain genomes, with each node representing a strain associated with its corresponding collection date. The infectious period, μ, is between seven and ten days [52,53]; edges with day differences of less than eleven days can be considered direct transmissions between the hosts. For an edge (*u*,*v*), the collection dates of *u* and *v* are ϕ(u) and ϕ(v) respectively. Then, |ϕ(u)−ϕ(v)| is the day difference of the edge (*u*,*v*). So, any edge of VEG having |ϕ(u)−ϕ(v)|≤μ can be considered as direct transmission between the two corresponding hosts.

## Results

### Count of unknown nucleotides

**Fig 5:**
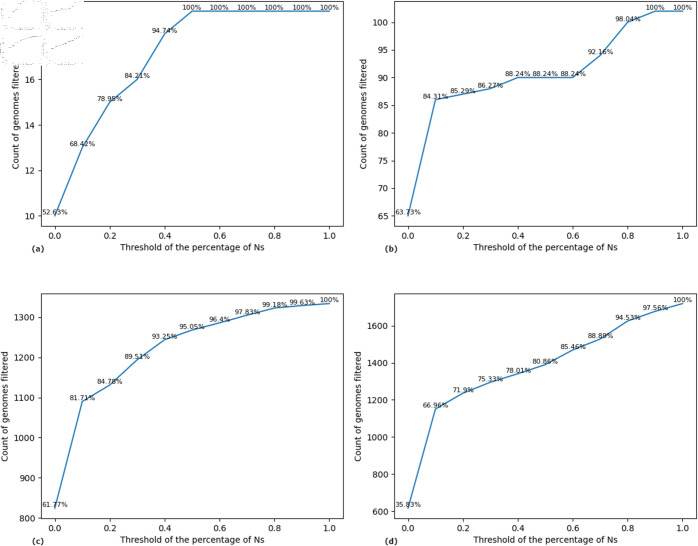
Count of genomes filtered based on the percentage of Ns, τ, in the genomes. The x-axis shows the threshold values and the y-axis shows the count of filtered genomes. The plots are of (a) Somalia, (b) Bhutan, (c) Iran, and (d) Nepal data sets.

As the Materials and Methods section outlines, the proportion of unknown nucleotides is a critical filtering criterion during data set preprocessing. Empirical analysis across multiple experiments revealed that complete, high-coverage genomes obtained from GISAID generally contain less than 1% of Ns. To quantify the impact of this parameter, we evaluated how varying the threshold value τ influences the number of genomes retained after filtering. Fig 5 presents the relationship between the threshold τ and the resulting genome counts for data sets from Somalia, Bhutan, Iran, and Nepal. For our study, we adopted a filtering threshold of τ=0, thereby including only genomes with no Ns in their sequences to maximize data quality and ensure the accuracy of downstream analyses.

**Fig 6:**
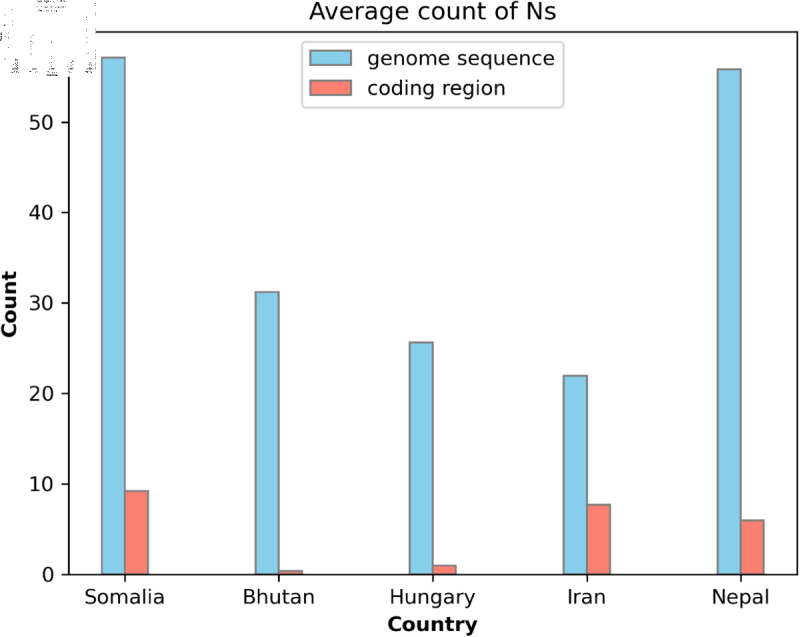
Average count of Ns in the genome sequences vs the coding regions in five data sets.

A substantial difference was observed in the number of unknown nucleotides between complete genome sequences and their corresponding coding regions. While we adopted a threshold of τ=0 for graph construction—ensuring that only genomes with no Ns were included—this threshold remains configurable based on the requirements of a given analysis. Under the τ=0 condition, the absence of Ns in the complete genome sequence guarantees that the extracted coding regions are also free of unknown nucleotides. Fig 6 compares the average number of Ns in the complete genome sequences and their associated coding regions across data sets from Somalia, Bhutan, Hungary, Iran, and Nepal. The results consistently indicate that coding regions contain significantly fewer Ns than their corresponding full-length genome sequences.

### Variant evolution graph

**Fig 7:**
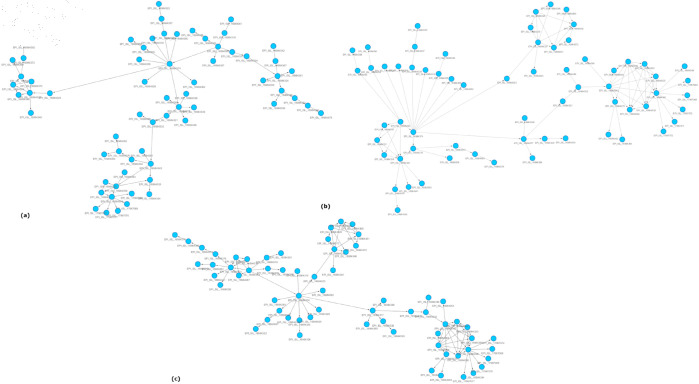
(a) ${VEG}_{S}$ (b) ${VEG}_{E}$, and (c) ${VEG}_{P}$ of Bhutan data set (graph viewed using Cytoscape 3.10.2).

We aim to build a directed and weighted VEG based on the mutational distance computed using sourmash, pyani, and edit distance. As a result, our method produces three graphs: $VEG_{S},VEG_{P},VEG_{E}$, respectively. The variant evolution graphs: $VEG_{S}$, $VEG_{P}$, and $VEG_{E}$ for the Bhutan data set are shown in Fig 7. All the *VEG*s of the other data sets (Somalia, Nepal, Iran, and Hungary) are provided in the Supporting information (S1 fig, S2 fig, S3 fig, S4 fig, S5 fig, S6 fig, S7 fig, S8 fig, S9 fig, and S10 fig). For each distance matrix mentioned, our algorithm gives these outputs: evolution history log, the adjacency matrix of VEG, lineage information, and edge list, which can be used directly to view VEG using Cytoscape.

The circular layout of Cytoscape [54] is a powerful visualization technique that circularly arranges nodes, providing an intuitive way to analyze network structures. In the context of viral evolution, VEG often consists of strongly connected subgraphs and tree-like structures. The circular layout enhances interpretability by organizing these components systematically, ensuring that clusters of variants and their relationships are visually distinguishable. The tree-like evolution paths, representing clear parent-child relationships, can be observed as radial extensions from the core of the circular structure, allowing for an easy distinction between ancestral and descendant variants. The strongly connected subgraphs (SCS) provide crucial insights into viral evolution and transmission dynamics. One key interpretation of an SCS in VEG is recombination, where different strains exchange genetic material, forming complex bidirectional relationships. Convergent evolution may form SCS in VEG, which occurs when different viral strains independently acquire similar mutations due to selective pressures, such as immune escape or antiviral resistance, leading to distinct lineages evolving functionally or structurally similar traits. Some SCSs may indicate high mutation flux regions, highlighting genetic hotspots where rapid mutations accumulate due to host immune responses or polymerase errors. Persistent, strongly connected subgraphs can also reflect circulating variants that continuously evolve, adapting to host immunity, vaccination, or drug treatments, which is particularly relevant for RNA viruses like SARS-CoV-2. Furthermore, an SCS may capture intra-host viral diversity within individual hosts, where quasispecies evolve dynamically under immune pressure before transmission. From an epidemiological perspective, SCSs can help identify super-spreading events or dense transmission networks. Analyzing these structures enables researchers to infer recombination, parallel evolution, mutation hotspots, and significant transmission pathways, ultimately contributing to a better understanding of viral adaptation and outbreak dynamics.

### Comparison between phylogenetic tree and VEG

The Variant Evolution Graph (VEG) and a phylogenetic tree represent fundamentally different approaches to modeling viral evolution, each with unique strengths and limitations. A phylogenetic tree is a hierarchical, tree-like structure where all branches converge to a common root, representing a shared ancestor. Each internal node typically represents a common ancestor, and the edges denote evolutionary paths leading to the current variants at the leaf nodes. The tree structure enforces a strict, branching relationship, with a single lineage at each split, implying that each variant evolves from only one parent.

**Fig 8:**
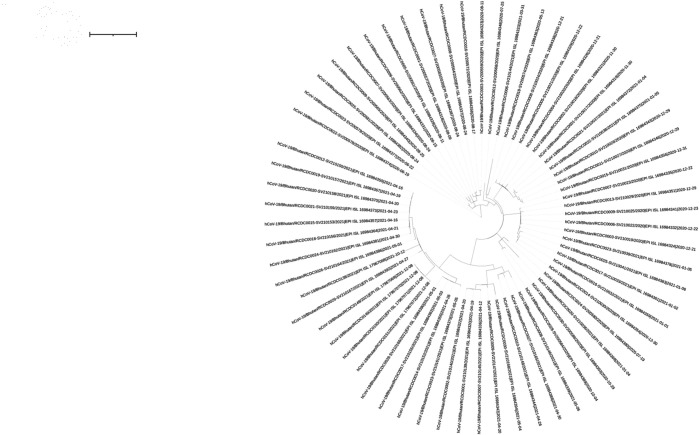
A maximum likelihood phylogenetic tree of the Bhutan data set.

We constructed a maximum likelihood phylogenetic tree using RAxML [55] to assess evolutionary relationships among the sequences of the Bhutan data set, shown in Fig 8, S11 fig, S12 fig, S13 fig, and S14 fig. The alignment of these sequences was used as input for phylogenetic inference under the GTRGAMMA model, which accounts for site-specific rate heterogeneity using a gamma distribution. Using the rapid bootstrap algorithm, we conducted 100 bootstrap replicates to evaluate branch support.

In contrast, the Variant Evolution Graph (VEG) (as seen in Fig 7) is a directed acyclic graph (DAG) that allows for more flexibility in representing evolutionary relationships. Unlike the tree, VEG does not require a single common ancestor and permits a variant to have multiple parent nodes. This structure reflects the complexity of viral evolution, where recombination events, convergent evolution, or multiple mutation pathways can lead to the same variant. The edges in VEG represent mutational distances and evolutionary direction, while the nodes correspond to variants linked to their hosts. VEG provides a richer and more interconnected view, making it ideal for capturing complex mutation pathways that phylogenetic trees may oversimplify or overlook.

The applications of these models further illustrate their complementary nature. Phylogenetic trees are ideal for understanding macro-evolutionary trends over long timescales, providing insights into the evolutionary history and relationships of viral lineages. However, VEG is better suited for real-time epidemic monitoring and localized outbreak analysis by directly integrating temporal and geographic data into its structure.

The Variant Evolution Graph (VEG) and phylogenetic trees fundamentally differ in structure, making direct structural comparisons impractical. Due to their inherent differences, structural metrics used for tree comparisons, such as Robinson-Foulds distance, do not apply to VEG. However, to provide a quantitative evaluation of computational efficiency, we benchmarked the runtime of both approaches (Table 3). We measured the execution time required to construct VEG and phylogenetic trees on the same data set, allowing for a direct performance comparison. The Maximum Likelihood (ML) approach is the slowest method in our comparison, primarily due to the high similarity of SARS-CoV-2 viral genomes. Since ML relies on multiple sequence alignment and probabilistic tree inference, it must evaluate numerous nearly identical topologies, making optimization computationally expensive. The minimal genetic variation among viral genomes further slows the process, as ML must search a vast space of possible trees with little distinguishing information. Among the VEG methods, edit distance also uses MSA to compute nucleotide differences between variant genomes, providing high accuracy while being computationally more efficient than ML and pyani. Pyani, which relies on BLAST for computing ANI, is slower than edit distance due to the overhead of sequence alignment. Sourmash, which employs a MinHash-based approach to compare genome sketches, is the fastest, sacrificing some precision for scalability. Since VEG methods avoid full tree inference, they offer significant computational advantages, making them more suitable for analyzing large viral data sets.

**Table 3:** Benchmarking the runtime of both approaches: VEG (edit distance, pyani, sourmash) and phylogenetic tree.

	edit distance (s)	pyani (s)	sourmash (s)	raxml (s)
Somalia	4.4	8.6	7.283	6.9
Bhutan	83.4	154.5	10.4	169.8
Hungary	3794.5	18229.5	147.7	3800.1
Iran	12702.8	39049.3	539.2	118607.5
Nepal	7080.3	25574.7	238.4	98604.0

### Comparison among sourmash, pyani, and edit distance

**Fig 9:**
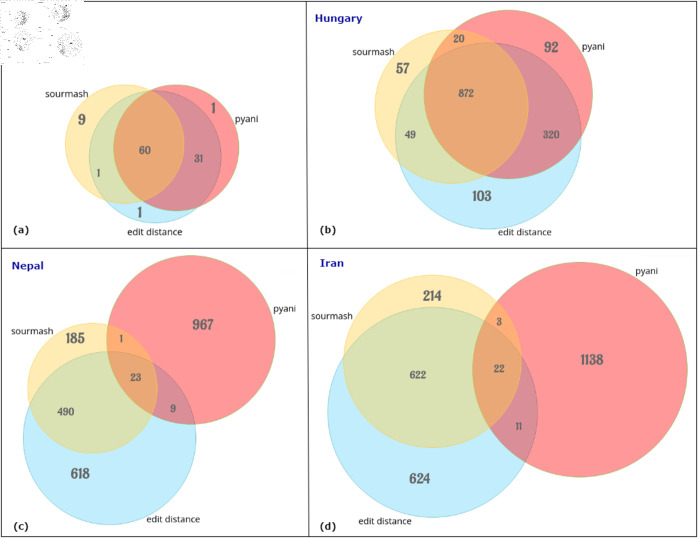
Venn diagrams showing parent-child relationships among the VEGs derived from sourmash, pyani, and edit distance. (a) Bhutan, (b) Hungary, (c) Nepal, and (d) Iran data sets.

The Venn diagrams in Fig 9 illustrate the similarities and differences in the parent-child relationships inferred from the Bhutan, Hungary, Nepal, and Iran data sets. The diagrams highlight that sourmash, pyani, and edit distance yield different parent-child relationships. Although sourmash and pyani estimate ANI, their inferred relationships differ significantly, as shown in the results. Edit distance, on the other hand, proves to be a more reliable and accurate measure of mutational distance since it directly reflects the nucleotide differences between two sequences. Additionally, sourmash and pyani often disagree on the ANI values among variant genomes. In contrast, edit distance emerges as a more promising method for measuring mutational distance, leading to more consistent and accurate results compared to sourmash and pyani.

### Disease transmission network

**Fig 10:**
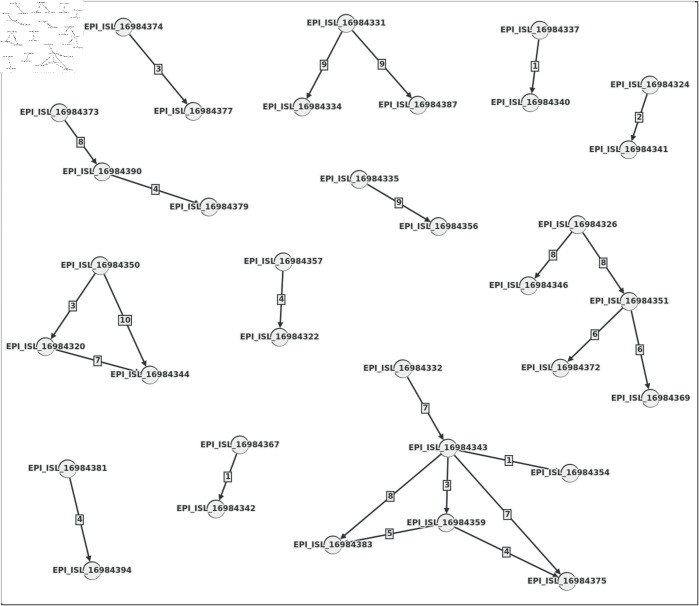
The DTN is inferred from the VEG of the Bhutan data set (edit distance). Here, the nodes are the hosts, and the edges represent the direction and day differences of the inferred transmissions.

Fig 10 illustrates the inferred Dynamic Transmission Network (DTN) of the Bhutan data set. Ideally, the DTN should be connected during a pandemic if all patients are tested promptly and their variants are sequenced and stored in a central database. However, the DTN of Bhutan, as shown in Fig 10, is disconnected, likely reflecting the circumstances surrounding the COVID-19 pandemic. If a substantial portion of the samples in GISAID had met the criteria described in the Data Set section, the Variant Evolution Graph (VEG) and its corresponding DTN would have been more comprehensive, encompassing a more significant number of variants and enabling the inference of more transmissions. These limitations highlight the importance of larger, more diverse data sets for accurate and detailed transmission network analyses.

### Identifying superspreaders

Several studies have indicated that 20% of the host population has the potential to cause 80% of transmission occurrences, a pattern known as the 80/20 rule [56–60]. Understanding the role of superspreaders might lead to more efficient disease outbreak containment and more accurate epidemic modeling [61].

In the DTN mentioned earlier, the nodes are the hosts, among which some superspreaders exist, whose out-degrees can be found. For a transmission network, we list the count of out-degrees of all the nodes and, sorting the list in descending order, we separate it into two parts: higher-degree set with the topmost 20% of the degrees and lower-degree set with the rest. According to the 80/20 rule, the nodes of this higher-degree set are responsible for 80% of the transmission, which means the nodes of this set have 80% of the total out-degrees.

Table 4 shows the statistics of Bhutan, Hungary, Iran, and Nepal data sets where the $80^{\text{th}}$ percentile is 1.0, 2.0, 1.0, and 1.0, respectively. This means all the out-degrees higher than these values belong to the higher 20%. Partitioning the out-degree lists of these data sets into the higher-degree and lower-degree sets, we found out that for the Bhutan data set, 60% of the out-degrees belong to the higher-degree set, 80.37% for the Hungary data set, 90.71% for the Iran data set, and 78.33% for the Hungary data set, as shown in Table 5. Some percentages do not exactly follow the 80/20 rule, which can be explained by the limitations mentioned earlier.

**Table 4:** Statistics showing the degree distribution of the vertices in disease transmission networks of Bhutan, Hungary, Iran, and Nepal.

	Bhutan	Hungary	Iran	Nepal
**count**	34	318	624	326
**mean**	0.735294	1.361635	1.466346	0.990798
**std**	0.931237	2.573471	4.690637	2.140433
**min**	0.000000	0.000000	0.000000	0.000000
**20%**	0.000000	0.000000	0.000000	0.000000
**40%**	0.000000	0.000000	0.000000	0.000000
**50%**	0.500000	0.000000	0.000000	0.000000
**60%**	1.000000	1.000000	0.000000	1.000000
**80%**	1.000000	2.000000	1.000000	1.000000
**max**	4.000000	17.000000	62.000000	17.000000

**Table 5:** Percentages of the count of out-degrees in the higher-degree sets of Bhutan, Hungary, Iran, and Nepal data sets.

Country	Percentage
Bhutan	60
Hungary	80.37
Iran	90.71
Nepal	78.33

## Discussion and conclusion

The Variant Evolution Graph (VEG) represents a transformative approach to understanding viral evolution and disease transmission, offering a paradigm shift from traditional tree-based models to a more dynamic, flexible, and comprehensive framework. By capturing the complex web of mutational relationships, VEG transcends the limitations of strictly hierarchical phylogenetic trees. The SCS in VEG provides critical insights into viral evolution by capturing recombination events, parallel evolution, and mutation hotspots that are difficult to represent in traditional phylogenetic trees. The SCS could also reflect intra-host viral evolution, where multiple related variants arise and interact before transmission.

Regarding computational efficiency, sourmash, edit distance, pyani, and Maximum Likelihood (ML) approaches exhibit significant differences. Sourmash, which uses a MinHash-based *k*-mer The sketching approach is the fastest among the VEG methods, making it highly scalable for large data sets but slightly less precise. The edit distance approach calculates pairwise mutational distances using MSA and balances computational efficiency with accuracy, making it highly accurate but still faster than pyani. Pyani, which relies on BLAST-based ANI calculations, is slower than edit distance due to the overhead of sequence alignment. In contrast, the maximum likelihood approach is the slowest overall, as it requires MSA and probabilistic tree inference, making it computationally expensive for large data sets of highly similar viral genomes. The results demonstrate that VEG methods offer a computationally efficient and scalable alternative to phylogenetic analysis while capturing complex evolutionary relationships, such as recombination and strongly connected clusters often overlooked in tree-based methods.

In conclusion, the VEG framework offers a holistic and scalable platform that aligns with the complexities of viral evolution and modern epidemiological challenges. Integrating genomic, temporal, and geographic data provides an unparalleled lens for studying the interplay of evolutionary and epidemiological forces. The method we have developed is not limited to SARS-CoV-2 but is a versatile tool that can be applied to any viral outbreak. By embracing the graph-based paradigm, researchers and policymakers can gain deeper insights into pathogen evolution and transmission dynamics, ultimately enhancing our ability to respond to current and future pandemics.

## Supporting information

S1 Fig VEGs of the Somalia data set (a) V EG_E_, (b) VEG_P_, and (c) VEG_S_
(PDF)

S2 Fig VEG_E_ of the Nepal data set. (PDF)

S3 Fig VEG_P_ of the Nepal data set. (PDF)

S4 Fig VEG_S_ of the Nepal data set. (PDF)

S5 Fig VEG_E_of the Hungary data set. (PDF)

S6 Fig VEG_P_ of the Hungary data set. (PDF)

S7 Fig VEG_S_ of the Hungary data set. (PDF)

S8 Fig VEG_E_of the Iran data set. (PDF)

S9 Fig VEG_P_ of the Iran data set. (PDF)

S10 Fig VEG_S_ of the Iran data set.
(PDF)

S11 Fig A maximum likelihood phylogenetic tree of the Somalia data set. (PDF)

S12 Fig A maximum likelihood phylogenetic tree of the Nepal data set. (PDF)

S13 Fig A maximum likelihood phylogenetic tree of the Hungary data set. (PDF)

S14 Fig A maximum likelihood phylogenetic tree of the Iran data set. (PDF)
