# Supplementary figures and images for: Variant evolution graph: Can we infer how SARS-CoV-2 variants are evolving?
Source: PLoS One. 2025 Jun 9;20(6):e0323970. doi: 10.1371/journal.pone.0323970 (PMC12148141; doi:10.1371/journal.pone.0323970)

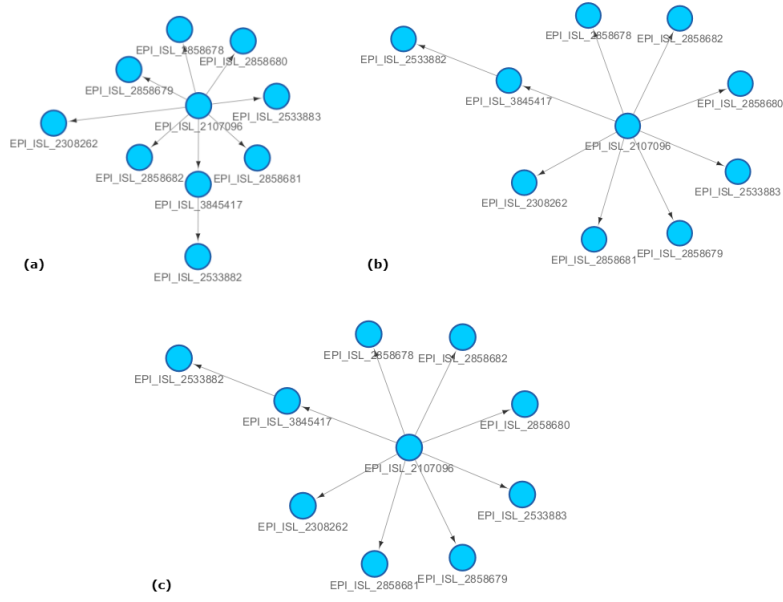

Figure 1: (a)  $VEG_E$ , (b)  $VEG_P$ , and (c)  $VEG_S$  of the Somalia data set.

Supplement: S1 Fig — (a) V EGE, (b) VEGP, and (c) VEGS (PDF) [file pone.0323970.s001.pdf]

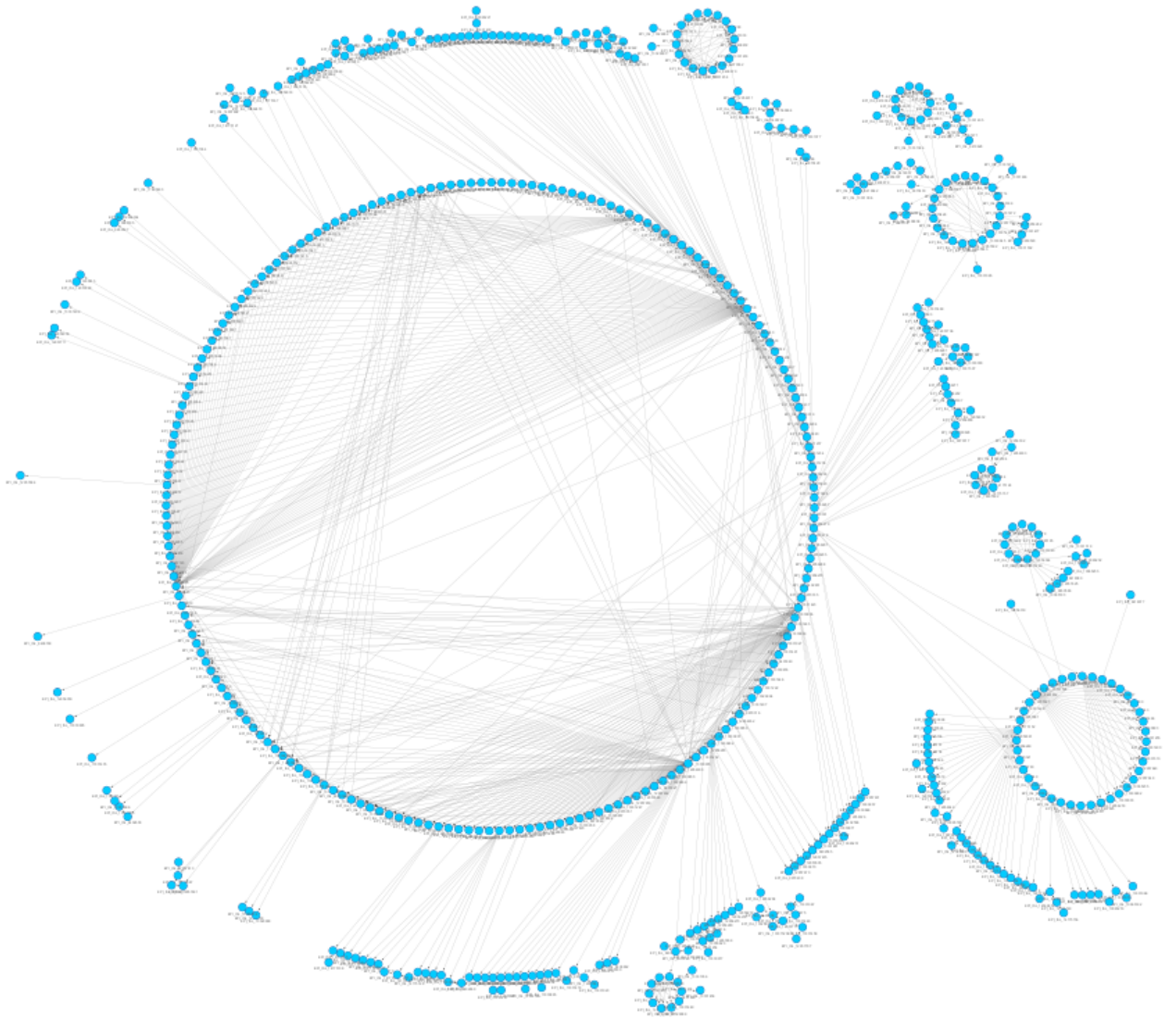

Figure 1:  $VEG_E$  of the Nepal data set.

Supplement: S2 Fig — (PDF) [file pone.0323970.s002.pdf]

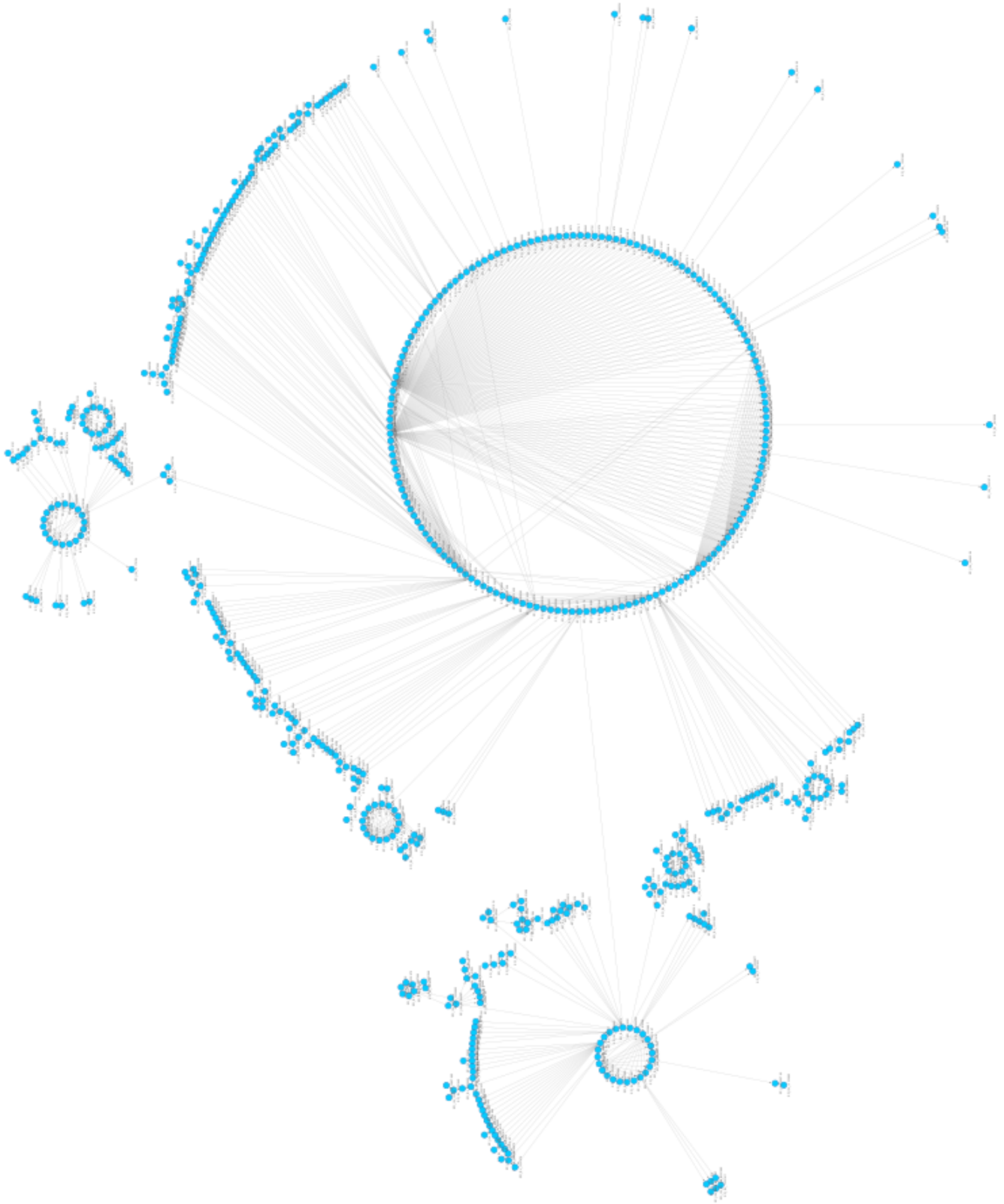

Figure 1:  $VEG_P$  of the Nepal data set.

Supplement: S3 Fig — (PDF) [file pone.0323970.s003.pdf]

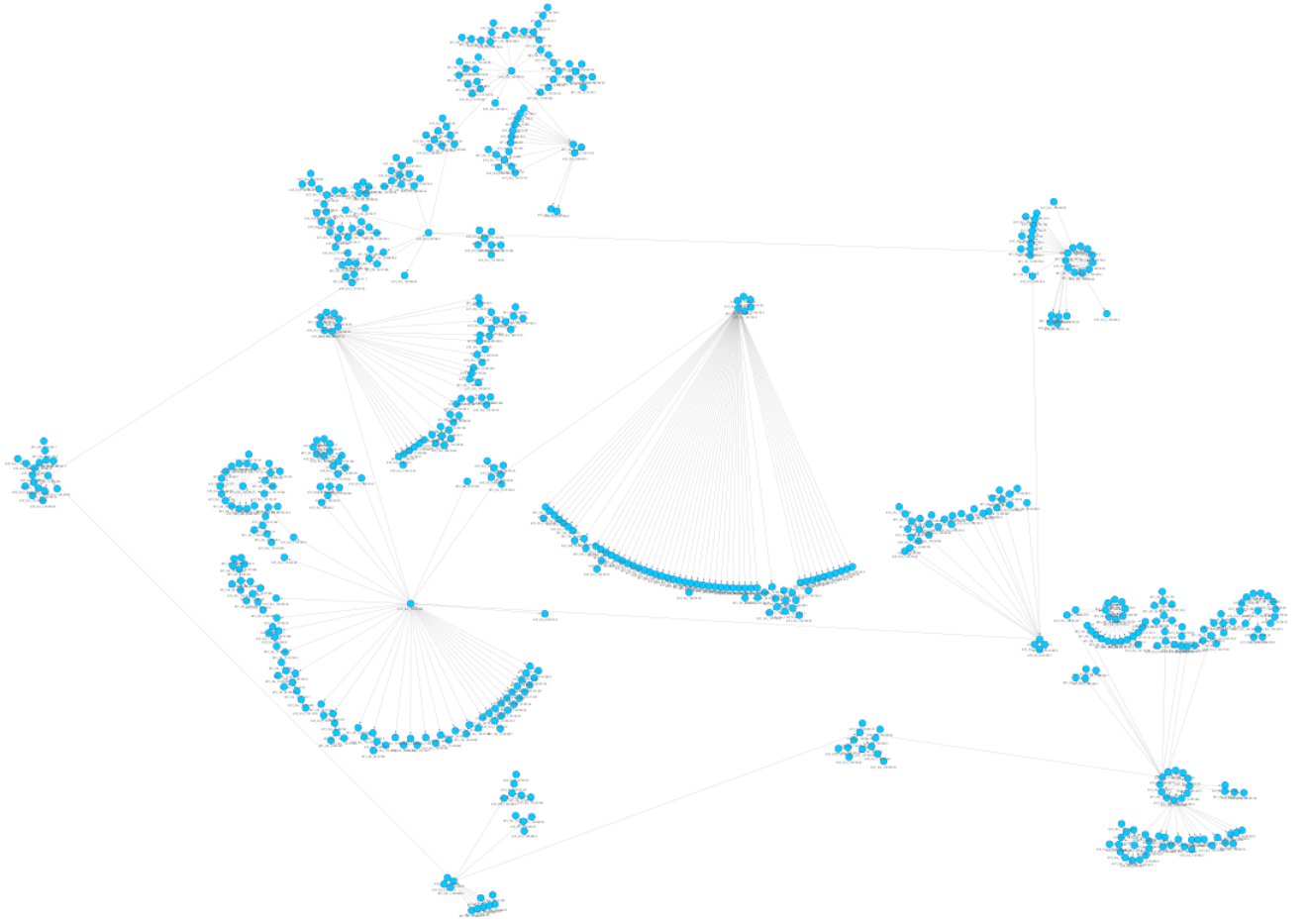

Figure 1:  $VEG_S$  of the Nepal data set.

Supplement: S4 Fig — (PDF) [file pone.0323970.s004.pdf]

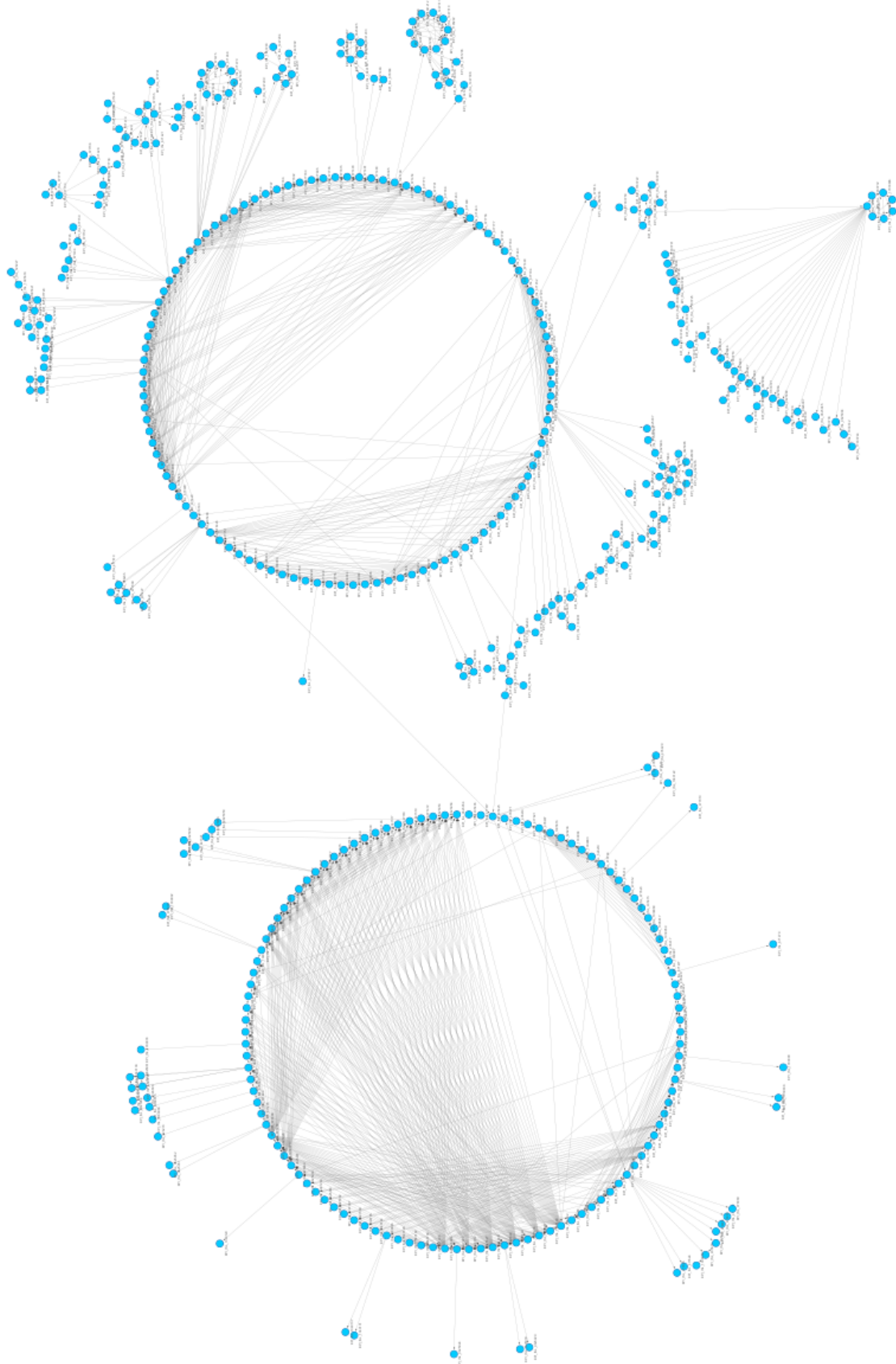

Figure 1:  $VEG_E$  of the Hungary data set.

Supplement: S5 Fig — (PDF) [file pone.0323970.s005.pdf]

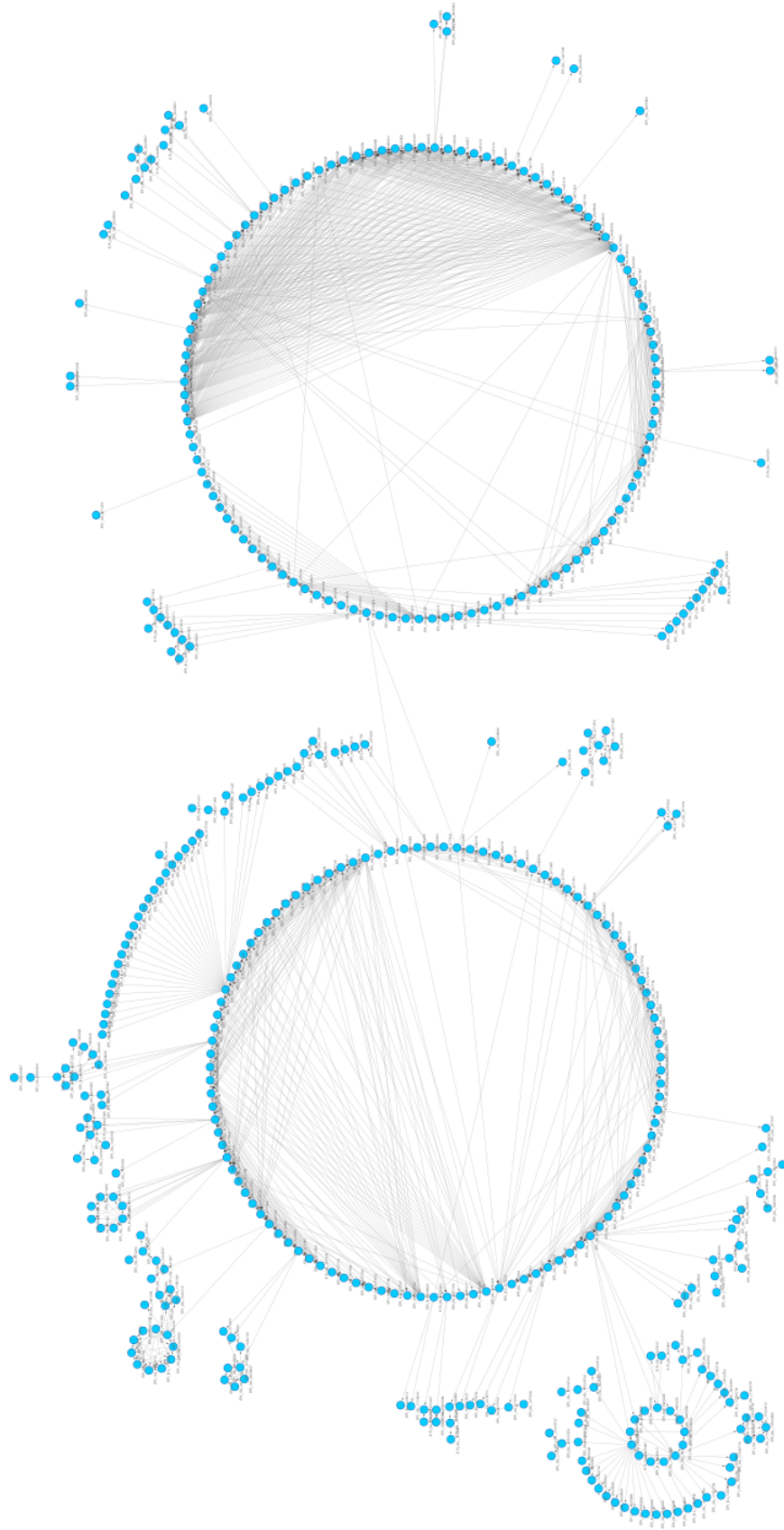

Figure 1:  $VEG_P$  of the Hungary data set.

Supplement: S6 Fig — (PDF) [file pone.0323970.s006.pdf]

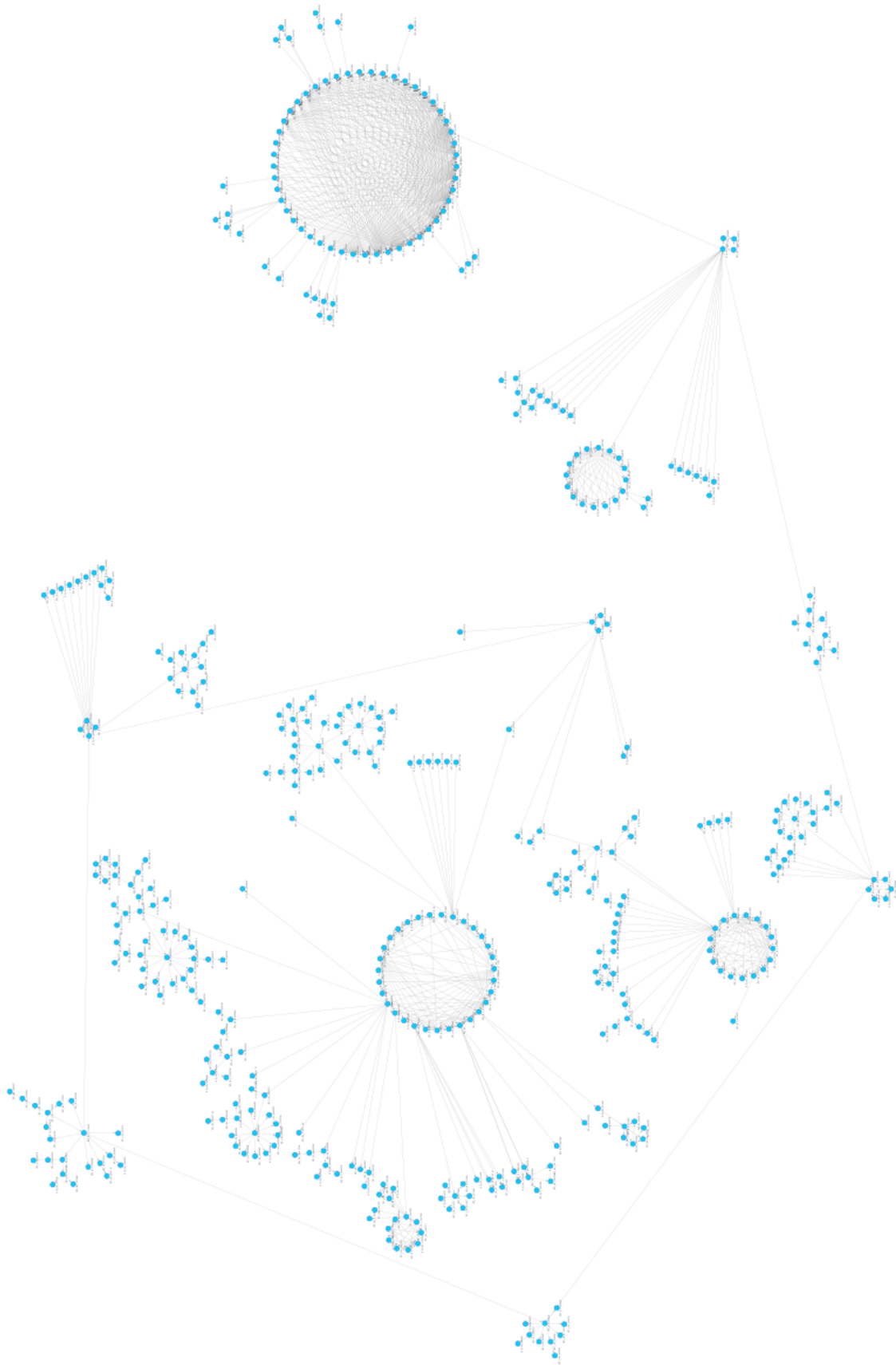

Figure 1:  $VEG_S$  of the Hungary data set.

Supplement: S7 Fig — (PDF) [file pone.0323970.s007.pdf]

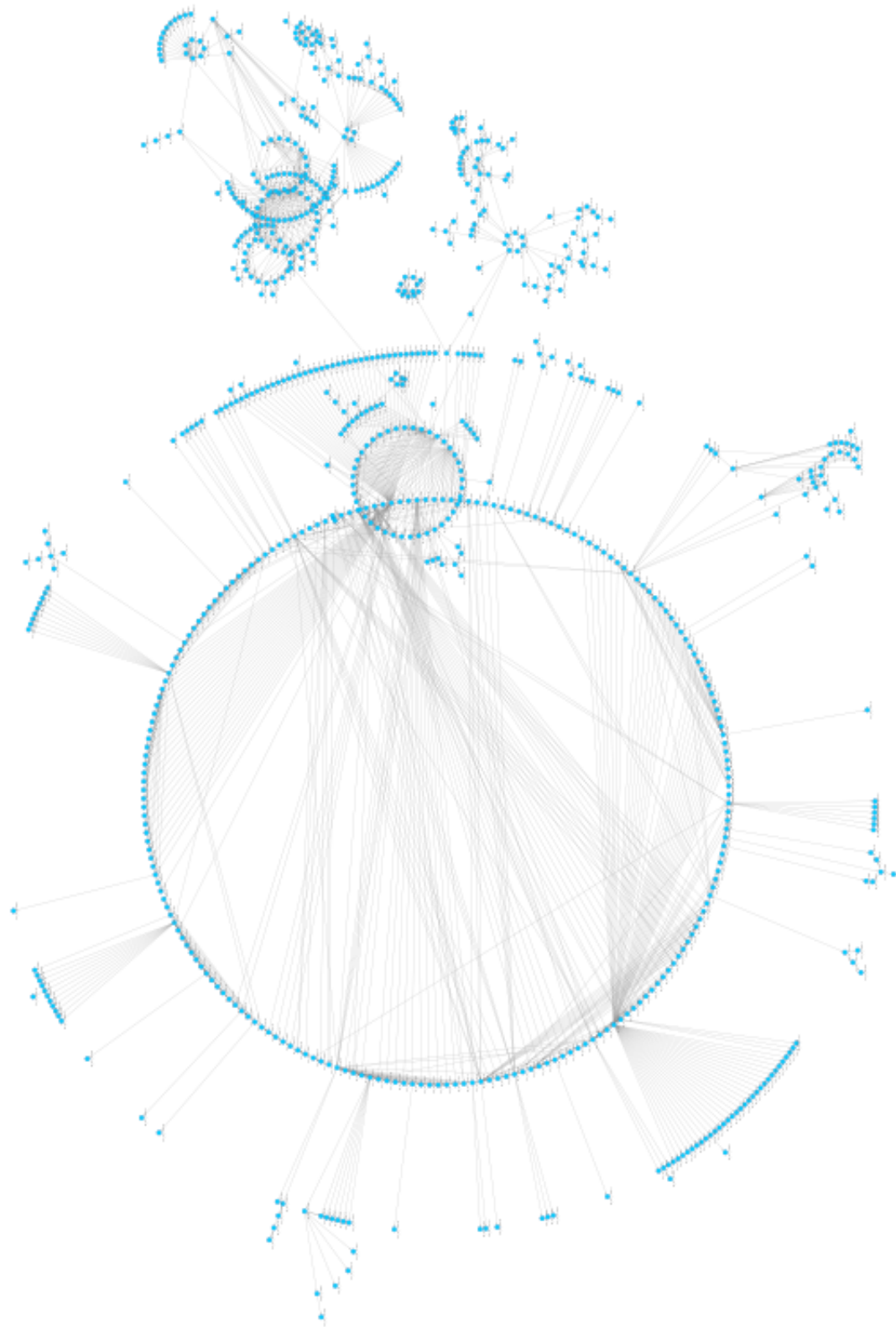

Figure 1:  $VEG_E$  of the Iran data set.

Supplement: S8 Fig — (PDF) [file pone.0323970.s008.pdf]

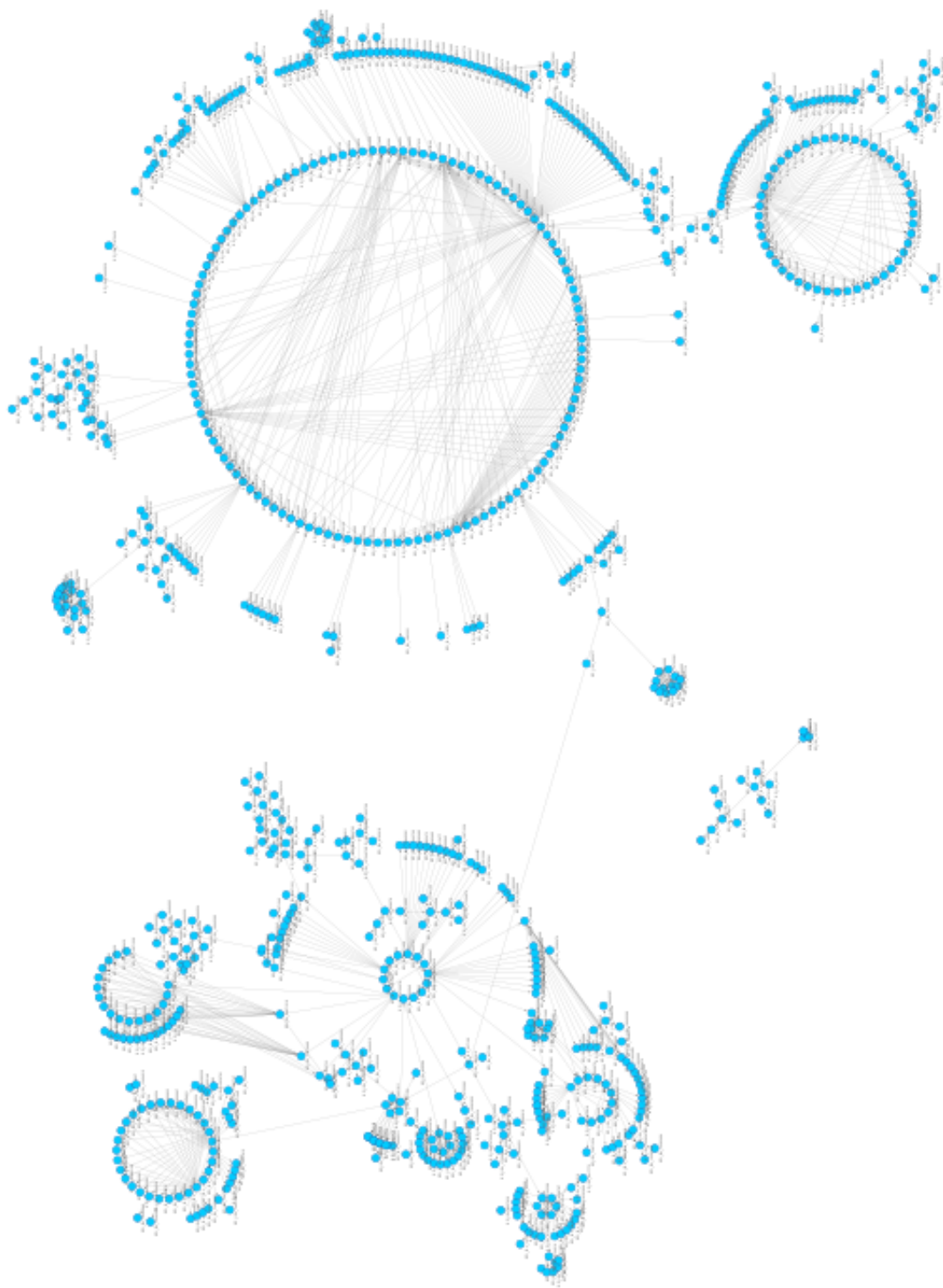

Figure 1:  $VEG_P$  of the Iran data set.

Supplement: S9 Fig — (PDF) [file pone.0323970.s009.pdf]

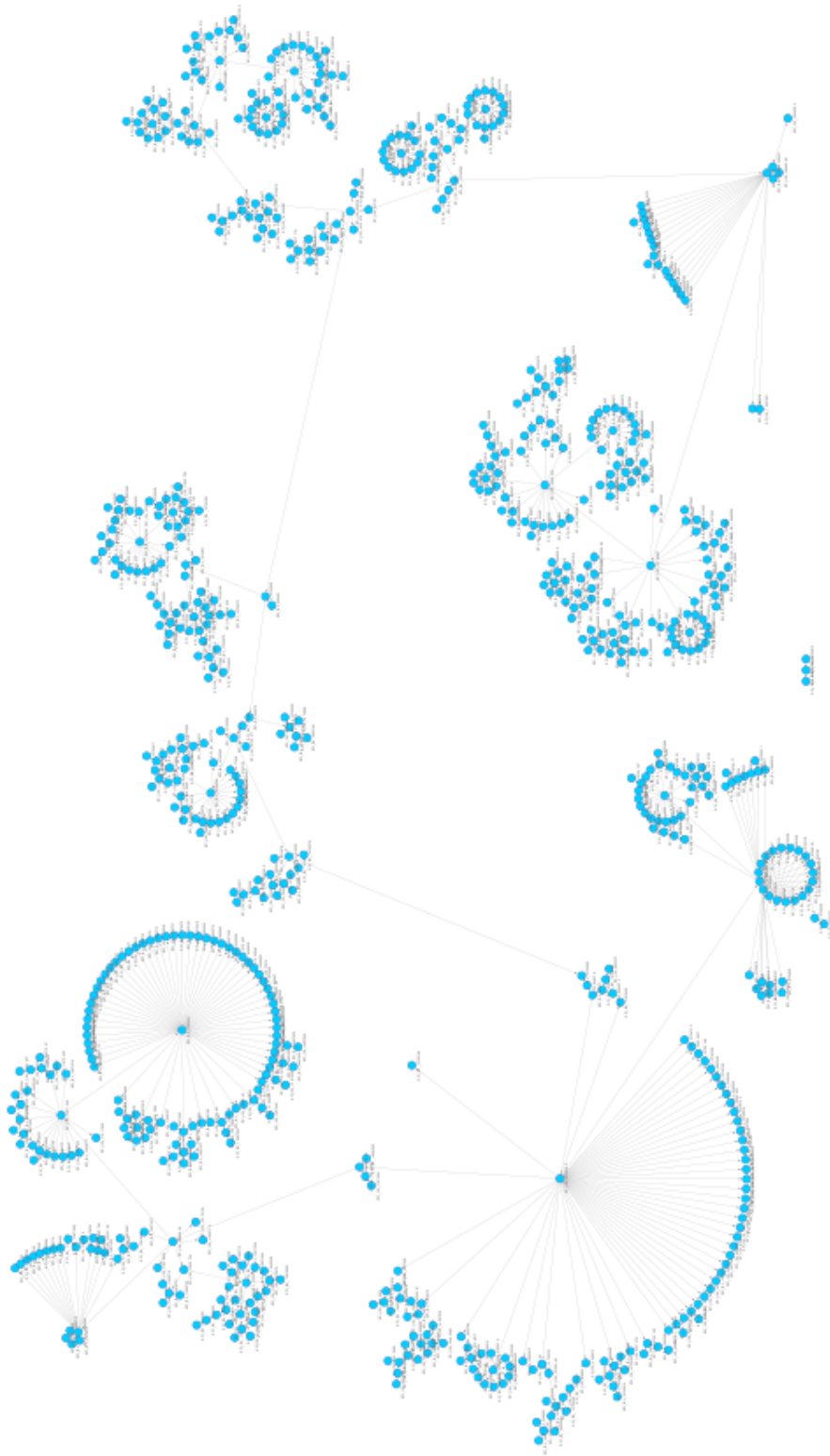

Figure 1:  $VEG_S$  of the Iran data set.

Supplement: S10 Fig — VEGS of the Iran data set. (PDF) [file pone.0323970.s010.pdf]

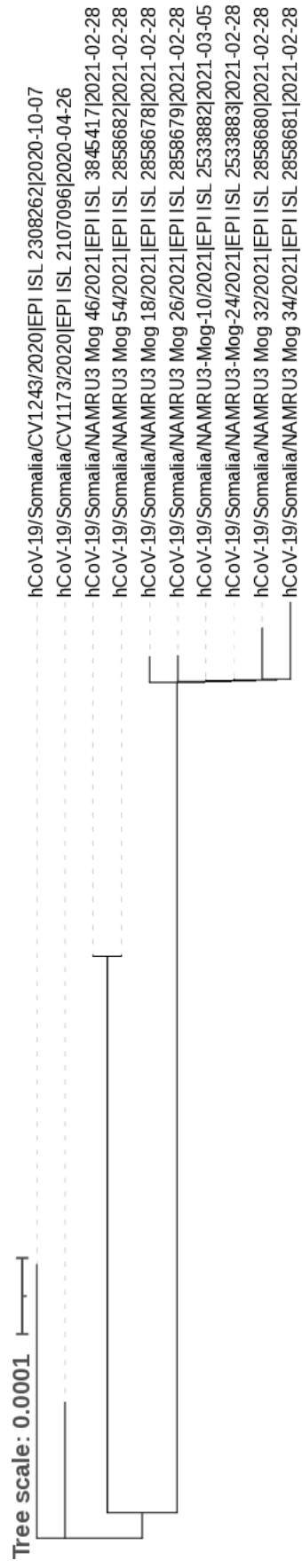

Figure 1: A maximum likelihood phylogenetic tree of the Somalia data set.

Supplement: S11 Fig — (PDF) [file pone.0323970.s011.pdf]

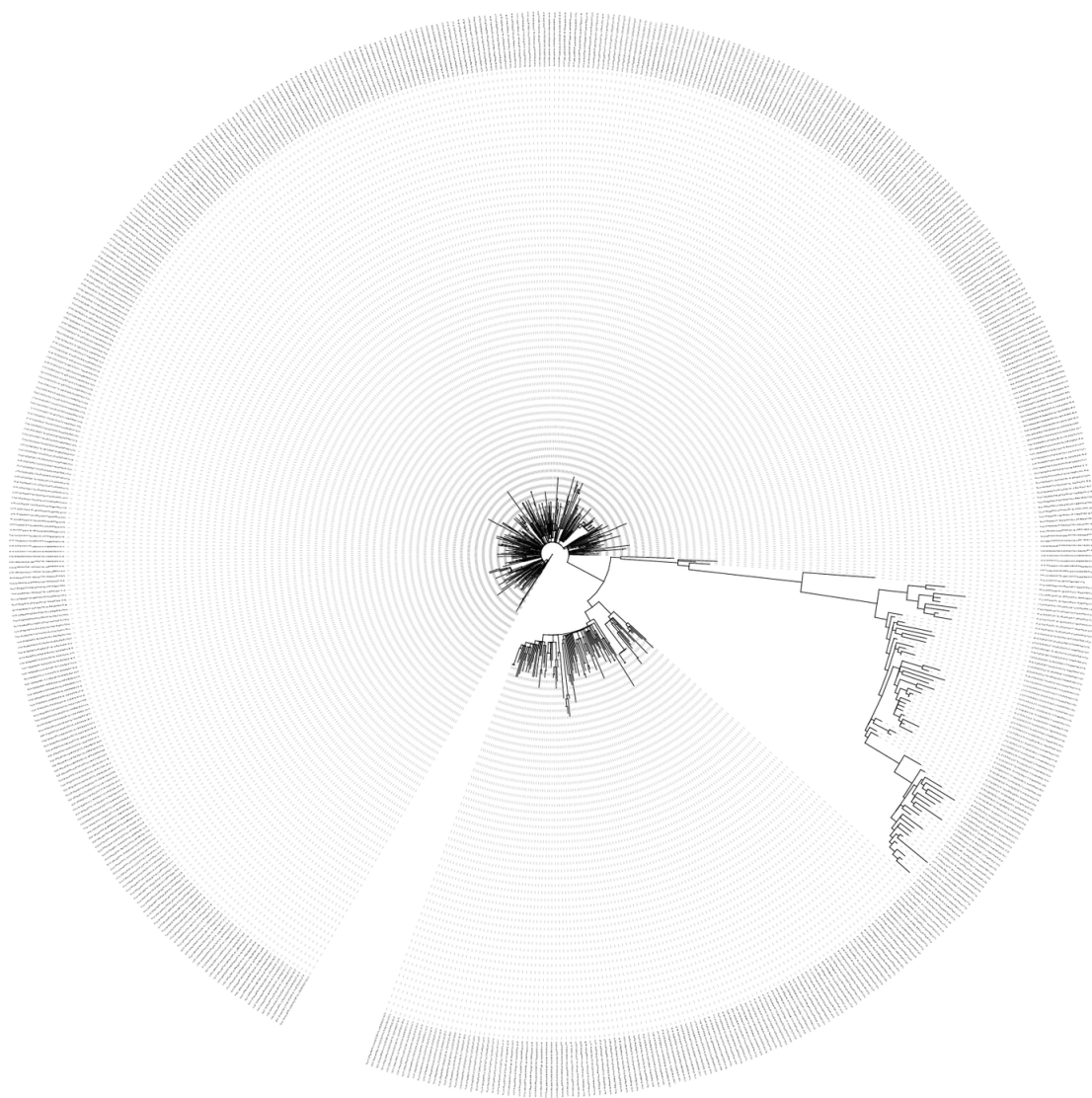

Figure 1: A maximum likelihood phylogenetic tree of the Nepal data set.

Supplement: S12 Fig — (PDF) [file pone.0323970.s012.pdf]

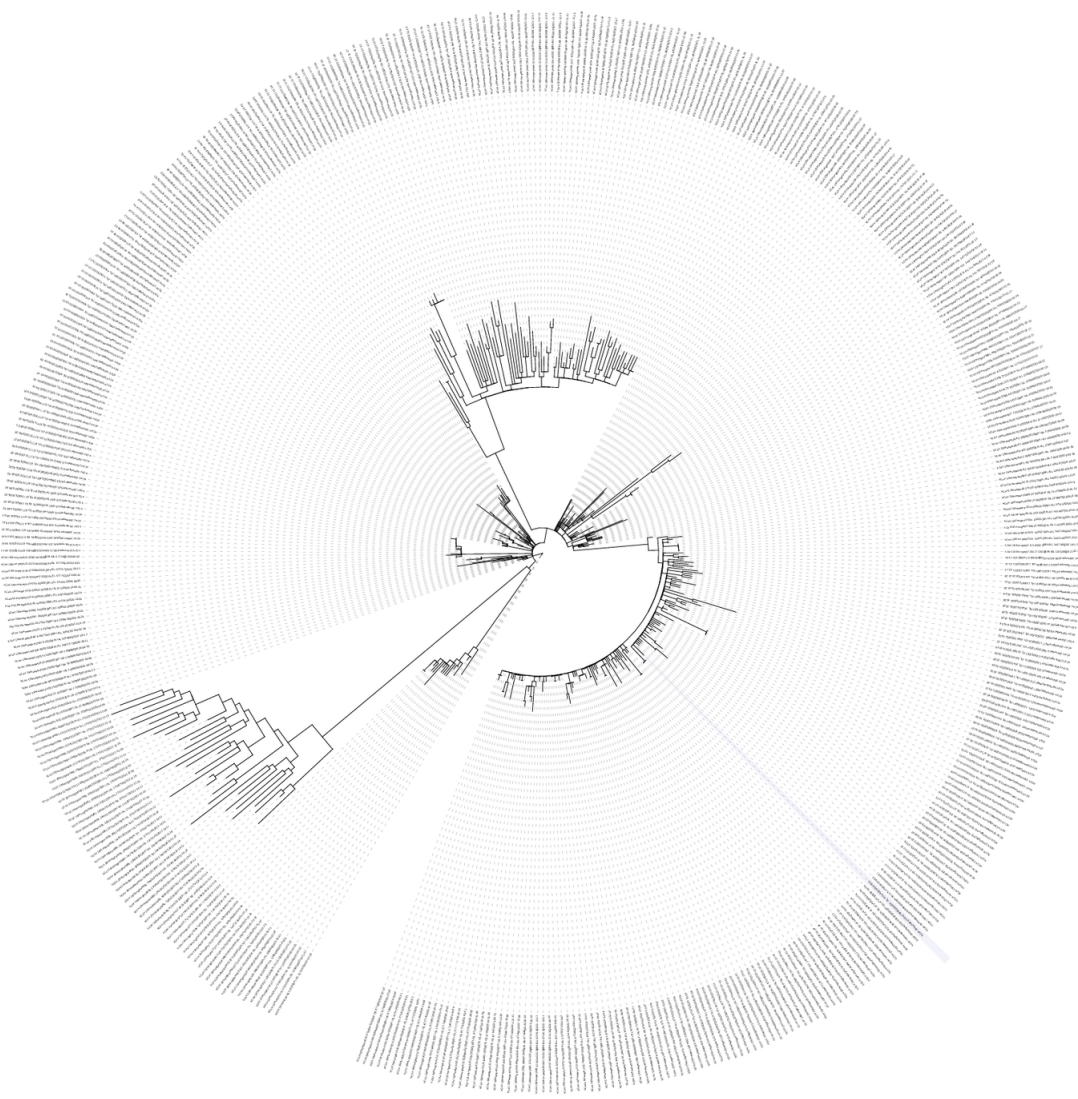

Figure 1: A maximum likelihood phylogenetic tree of the Hungary data set.

Supplement: S13 Fig — (PDF) [file pone.0323970.s013.pdf]

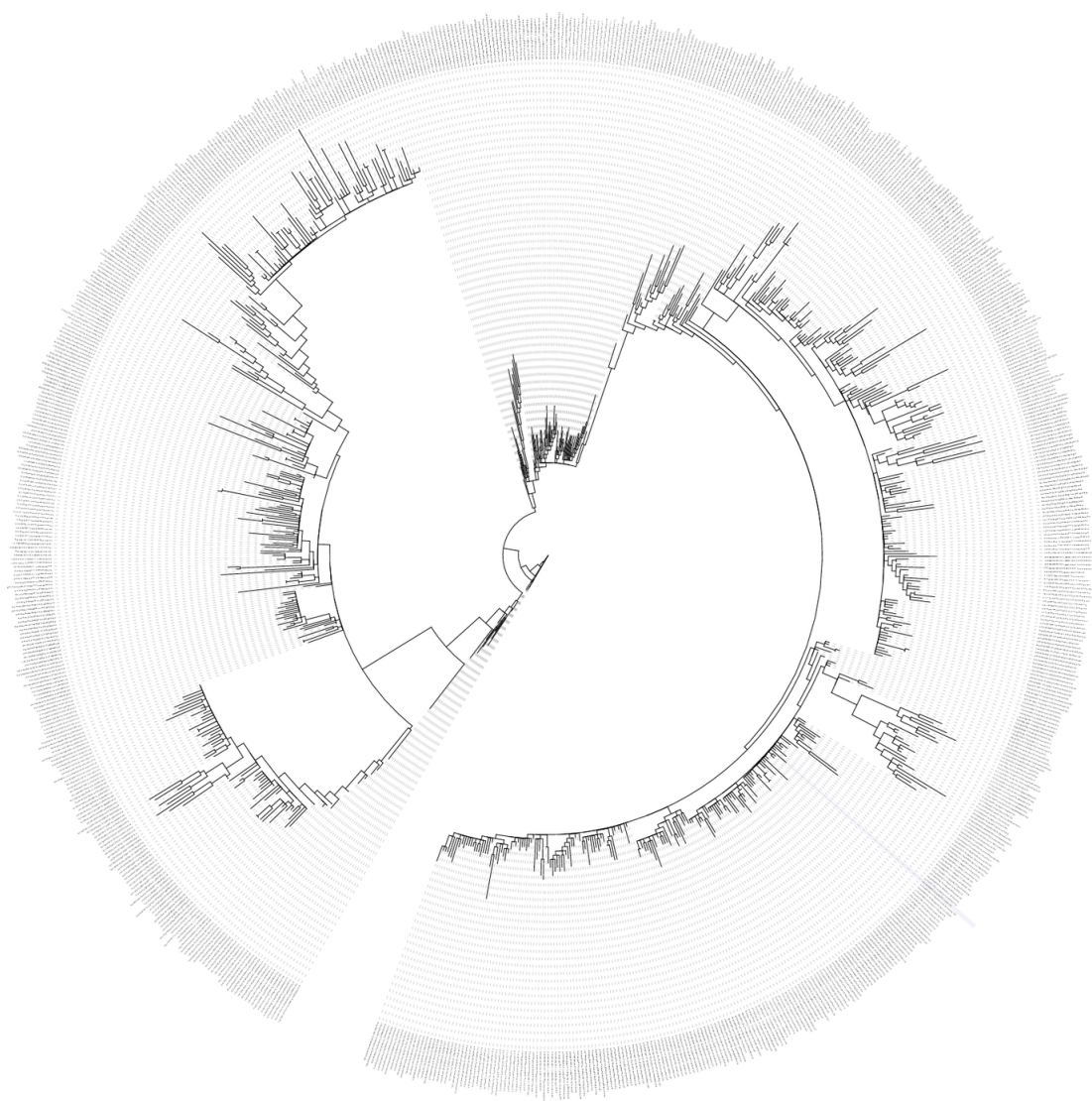

Figure 1: A maximum likelihood phylogenetic tree of the Iran data set.

Supplement: S14 Fig — (PDF) [file pone.0323970.s014.pdf]
